# Immune-inflammatory, neuroplastic, and epigenetic effects of electroconvulsive therapy in mood disorders: an overview of recent studies

**DOI:** 10.3389/fpsyt.2025.1577530

**Published:** 2025-09-03

**Authors:** Rafael Batista João, João Paulo Rocha Pereira Toiansk de Azevedo, Dácio Almeida Pereira, Paulo César Ragazzo, Paulo Maurício de Oliveira

**Affiliations:** ^1^ Neurophysiology Department, Goiânia Neurological Institute, Goiânia, Goiás, Brazil; ^2^ Psychiatry Department, Goiânia Neurological Institute, Goiânia, Goiás, Brazil; ^3^ Psychiatry Department, Federal University of Goiás, Goiânia, Goiás, Brazil

**Keywords:** electroconvulsive therapy, ECT, neuroinflammation, inflammation, neuroplasticity, genetics, epigenetics

## Abstract

**Background:**

Electroconvulsive therapy (ECT) remains an effective intervention for severe and treatment-resistant mood disorders, particularly major depressive disorder (MDD) and bipolar disorder (BD). While traditionally linked to neurotransmitter modulation, recent research suggests that ECT exerts broader biological effects. Currently, there is a necessity for identifying factors that could support a more accurate selection of individuals, predict their therapeutic response, and help investigate evidence of possible neuroplastic effects of this technique. In this setting, many studies have been published in the last few years, aiming to identify potential biomarkers by understanding immune-inflammatory, structural, and cellular mechanisms and their correlations with clinical outcomes post-ECT.

**Methods:**

We searched PubMed, Embase, Scopus, Web of Science, PsycINFO, and Cochrane Library for studies published between 2020 and 2025. Studies were selected based on their relevance to inflammatory, immune, structural, and cellular mechanisms associated with ECT.

**Results:**

Twenty-six studies were included. The main results reported post-ECT reductions in inflammatory markers, including C-reactive protein, interleukin-6, and tumor necrosis factor-alpha, or suggested a biphasic trajectory, with an initial transient immune activation preceding inflammatory partial resolution. Noteworthy differences were related to age, as younger patients showed more favorable immune adaptability in comparison with older individuals, who demonstrated elongated inflammatory activity. Neuroplastic changes following ECT were observed, including increased hippocampal neurogenesis, enhanced brain-derived neurotrophic factor expression, and structural changes in neuroimaging studies. Novel exploratory research on post-mortem analyses further confirmed the upregulation of neuroplasticity markers without evidence of sustained neuroinflammation. In addition, epigenetic mechanisms, particularly microRNA modulation following ECT, may induce long-term cellular reprogramming, potentially influencing treatment response. Moreover, one recent study suggested that elevated baseline levels of miR-223-3p may be a predictor of ECT response among treatment-resistant depression patients. Finally, a recent study exploring mitochondrial adaptations found that the interactions between mitochondrial DNA copy number, oxidative stress, and ECT remain inconclusive.

**Conclusion:**

Recent studies have expanded the understanding of ECT’s neuroinflammation effects and beyond, adding data on its interactions with immune, neuroplastic, and genetic mechanisms in human samples. Although many gaps still exist, these findings pave the way for further research that may improve outcomes of treatment-resistant mood disorders.

## Introduction

Electroconvulsive therapy (ECT) remains one of the most effective interventions for severe and treatment-resistant mood disorders, such as major depressive disorder (MDD) and bipolar disorder (BD) (1, 2). Meta-analytic data show that ECT achieves response and remission rates of 74.2% and 52.3% in MDD and 77.1% and 52.3% BD, respectively (3). While its clinical efficacy has been widely documented, the precise neurobiological mechanisms underlying ECT’s therapeutic effects are still under investigation (3, 4). ECT is one of the least understood biological treatments in psychiatry. Historically, ECT was found to act primarily through the modulation of neurotransmitter systems, particularly serotonin, dopamine, and gamma-aminobutyric acid (GABA) (4, 5). However, evidence suggests that its effects extend beyond synaptic neurotransmission, involving immune-inflammatory pathways, neuroplasticity, and genetic/epigenetic modifications (4, 6–8).

Neuroinflammation has been increasingly implicated in mood disorders, with elevated pro-inflammatory cytokines such as interleukin-6 (IL-6), tumor necrosis factor-alpha (TNF-α), and C-reactive protein (CRP) contributing to neuronal dysfunction and treatment resistance (9–11). ECT appears to modulate immune responses. However, research findings on this topic have been inconsistent, and many studies continue to explore how ECT influences inflammatory markers and clinical outcomes (12). Concomitantly, growing evidence suggests that ECT may support brain plasticity by increasing hippocampal volume, reshaping neural connections, and enhancing brain-derived neurotrophic factor (BDNF) expression (13). Recently, genetic and epigenetic changes, including DNA methylation shifts and altered microRNA expression, have been implicated in neural adaptation due to ECT modulation effects (8). Mitochondrial function has also been investigated in this setting, with studies linking ECT response to oxidative stress markers and mitochondrial DNA copy number (mtDNAcn) (12).

Understanding how ECT modulates immune-inflammatory pathways, neuroplasticity, and epigenetic processes is paramount to identifying biomarkers of response, improve patient selection, and guide the development of optimized neuromodulatory interventions. Despite recent advances, critical knowledge gaps persist. Thus, we performed this overview of recent findings of studies integrating ECT’s inflammatory, immune, neuroplastic, and genetic effects among individuals with mood disorders.

## Methods

We searched PubMed, Embase, Scopus, Web of Science, PsycINFO, and Cochrane Library databases to identify original studies published between January 2020 and April 2025 that investigated the effects of ECT on inflammatory, immune, genetic, and neuroplastic processes in mood disorders. The search strategy included terms and keywords related to “electroconvulsive therapy,” “inflammation,” “immune response,” “neuroplasticity,” “genetics,” “epigenetics,” “mood disorders”, “depression”, “depressive”, and “bipolar disorder”. In addition, we manually reviewed the references of all included articles and previous relevant reviews for additional studies.

Studies with human samples and correlating inflammatory parameters, molecular profiling, neuroimaging, post-mortem histopathology, or genetic markers were included. Eligible studies included participants diagnosed with major depressive disorder (MDD) or bipolar disorder (BD), as defined by standardized diagnostic criteria (e.g., DSM-IV, DSM-5, or ICD-10/11) (14–16). Biological measures encompassed inflammatory cytokines (e.g., IL-6, TNF-α, IL-1β, IL-10, CRP), hematological inflammatory indexes (e.g., NLR, PLR, SII), neurotrophic factors (e.g., BDNF), DNA methylation changes, microRNA expression, mtDNAcn, and post-mortem markers of neuroplasticity (e.g., DCX, STMN1) and glial activation (e.g., GFAP, Iba1), as well as structural neuroimaging biomarkers.

No restrictions regarding study design were applied; both observational and interventional studies were eligible. Preprint publications were excluded. Data extraction focused on sample characteristics, study design, primary findings, and clinical correlations. Key details of ECT protocols — including electrode placement, dosing strategies, pulse width, frequency, and total number of sessions — are summarized for each study in **Tables 1**–**3**.

**Table 1:** Main features of studies evaluating peripheral inflammatory and hematological biomarkers in the context of ECT response, cognitive adverse events, and symptom clusters.

Study, year	Sample	Study Design	ECT Protocol	Biomarkers Assessed	Key Findings	Clinical Outcomes	Conclusions & Implications
Du et al., 2024 (17)	38 adolescents with severe MDD (median age 15; 81.6% female); 29 healthy-controls (median age 15; 53.8% female)	Prospective cohort study	Bilateral ECT, Thymatron DGx, 6–8 sessions over two weeks	IL-1β, IL-6, IL-10	Post-ECT: IL-1β ↓, IL-6 ↓, IL-10 ↑; IL-1β and IL-6 reductions correlated with HDRS ↓	Significant reduction in HDRS scores post-ECT; IL-1β and IL-6 decreases correlated with symptom improvement.	ECT exerts anti-inflammatory effects in adolescents with severe MDD, potentially contributing to its antidepressant mechanism. Inflammatory marker shifts may serve as predictors of treatment response.
Mindt et al., 2020 (18)	12 patients with TRD (mean age 59.0; 58.3% female)	Prospective cohort study	Right unilateral ECT (Thymatron IV), 2–3x/week, mean of 10.6 ± 5.0 sessions; ketamine anesthesia	25 cytokines (including IL-1β, IL-6, IL-8, IL-17, IL-2R, RANTES, IP-10, MIP-1α, etc.) in CSF and serum	Post-ECT: CSF IL-5 ↑, IL-8 ↑, IP-10 ↑; IL-6 ↓ and IL-1β ↓ correlated with number of sessions; responders/remitters showed IL-17 ↓, MIP-1α ↓, RANTES ↓, IL-2R ↓; IP-10 ↑ was attenuated in remitters.	Significant HDRS reduction (mean from 29.9 to 9.0; p < 0.001); 83.3% responded, 41.7% remitted.	ECT alters CNS immune activation; reduction in CSF inflammatory cytokines is associated with antidepressant response. Findings support ECT’s impact on central (not only peripheral) inflammation.
Kruse et al., 2020 (19)	40 TRD patients (mean age 41.8; 55% female)	Prospective observational study	Right unilateral ultra-brief pulse ECT (5–6x seizure threshold), switching to bilateral if needed; mean 11.8 sessions	IL-8 (primary), IL-6, IL-10, TNF-α, CRP	Baseline IL-8 ↓ predicted response in females; IL-8 ↑ post-ECT correlated with HAM-D ↓ in females (β = –0.458; p = 0.03); no associations in males; no significant effects for IL-6, IL-10, TNF-α, or CRP.	50% response rate (≥50% HAM-D reduction); no remission data reported.	IL-8 may serve as a sex-specific biomarker of ECT response in females with TRD. Highlights the importance of considering sex-specific neuroinflammatory mechanisms in depression treatment personalization.
Ryan et al., 2022a (20)	86 patients with depression (mean age: 55.1; 61.6% female); 57 healthy controls (mean age: 51; 66.1% male)	Prospective case–control	Bilateral ECT, twice weekly; average of 8 sessions	Plasma CRP, IL-6, IL-10, and TNF-α	Baseline: IL-6 and TNF-α ↑ in patients vs. controls; No significant changes post-ECT.	Significant HAM-D24 score improvement. No correlations between inflammatory markers and cognitive outcomes.	Systemic inflammatory markers did not mediate ECT effects on mood or cognition.
Carlier et al., 2021a (21)	99 older adults with depression (mean age: 72.8; 66.7% female)	Prospective cohort study	Right unilateral ECT with titrated dosing; switch to bilateral after 6 sessions if no response; 2 sessions/week; median of 11 sessions (range 5–18)	Serum CRP, IL-6, IL-10, TNF-α	Post-ECT: CRP ↓ (d = –0.29), IL-6 ↓ (d = –0.13); IL-10 and TNF-α unchanged; higher baseline CRP predicted remission.	66.7% achieved remission (MADRS <10); 77.8% responded (≥50% MADRS reduction).	ECT did not significantly reduce inflammatory markers overall; higher pre-treatment inflammation (e.g., CRP) may predict better clinical response.
Loef et al., 2021 (22)	99 older adults with severe LLD (mean age: 72.8; 66.7% female)	Naturalistic longitudinal study	Right unilateral ECT (Thymatron System IV); switch to bilateral after 6 sessions if no response; 2 sessions/week; mean of 11.8 ± 5.53 sessions	Serum IL-6, TNF-α, BDNF; calculated IL-6/BDNF and TNF-α/BDNF ratios	Post-ECT: TNF-α/BDNF ratio ↑ associated with greater depression severity; higher TNF-α amplified the negative correlation between BDNF and MADRS; IL-6, TNF-α, and BDNF alone not associated.	Significant reduction in MADRS score: 33.6 → 9.6 (T0→T2) post-treatment; 83.9% responded, 71.1% remitted.	The interaction between TNF-α and BDNF may be implicated in the LLD pathophysiology and ECT response; BDNF effects are moderated by TNF-α levels.
Tian et al., 2021 (23)	40 patients with MDD (First-episode, drug-free, mean age 49.45; 50% males); 40 healthy controls (mean age 48.31; 55% female, and education matched	Cross-sectional case–control study	MECT, 12 sessions, 3/week; initiated with stable-dose antidepressants	NLRP3 inflammasome, IL-18, NF-κB	Baseline: NLRP3 ↑, IL-18 ↑, NF-κB ↑ in patients vs. controls; Post-MECT: further ↑ in all markers in MDD group only.	70% showed cognitive impairment (WMS-R score < 50) after MECT; this group showed significantly higher post-ECT levels of NLRP3, IL-18, and NF-κB compared to cognitively preserved participants (p < 0.05).	Inflammatory cytokines, such as NLRP3, IL-18, and NF-κB, may predict cognitive impairment risk after MECT. Elevated levels were significantly correlated with worse cognitive performance.
Ryan et al., 2022b (24)	62 patients with depression (mean age: 56.8; 66.1% female)	Prospective observational study	Bilateral ECT (frequency not specified); mean 7.6 sessions.	NLR, PLR, MLR, SII (pre-ECT)	Baseline: PLR and SII inversely correlated with depression severity; higher NLR, PLR, and SII predicted longer time to reorientation post-ECT.	Patients experienced variable disorientation post-ECT, with longer time to reorientation associated with higher baseline NLR, PLR, and SII; Depression outcome not reported.	Greater peripheral inflammatory burden may predict longer disorientation time post-ECT; suggests link between systemic inflammation and acute cognitive adverse events.
Belge et al, 2021 (25)	62 patients with major depressive episodes (mean age = 58.2; 77.4% female)	Prospective longitudinal study	Right unilateral brief-pulse ECT, 2 sessions/week; switched to bilateral if no response or faster effect needed; mean 11 sessions (range 2–27).	Serum IL-6	Post-ECT: IL-6; ↓ correlated with psychomotor improvement.	HDRS-17 decreased (24.6 → 8.7, p < 0.0001) post-treatment; psychomotor symptoms improved; IL-6 decrease correlated with psychomotor retardation improvement.	ECT-related improvement in psychomotor retardation may be associated with IL-6 decrease, supporting the role of inflammation in motor symptom improvement.
Carlier et al., 2021b (26)	97 older adults with unipolar depression (mean age: 73.1; 67% female)	Prospective cohort study	Right unilateral ECT with dose titration; bilateral after 6 sessions if non-response; median 11 sessions	CRP, IL-6, IL-10, TNF-α (baseline only)	Higher baseline TNF-α and IL-10 were associated with lower MMSE scores during treatment. Elevated baseline CRP predicted poorer post-ECT MMSE performance.	Cognitive performance affected during the ECT course, particularly among those with higher baseline levels of TNF-α and IL-10. Elevated baseline CRP was associated with lower MMSE scores post-ECT. IL-6 showed no significant associations.	Baseline inflammation markers may predict cognitive vulnerability to ECT in older adults.
Kirlioglu Balcioglu et al., 2025 (27)	156 patients with psychotic and mood disorders (mean age 36.09; 58.3% male)	Retrospective cohort study	Bilateral ECT, Thymatron System IV, 2–3 sessions/week, avg. 8.53 ± 2.05 sessions	CRP, CRP-albumin ratio, NLR, MLR, PLR, CAR	Post-ECT: CRP ↓, NLR ↓, MLR ↓, PLR ↓, CAR ↓	Significant reductions in BPRS and CGI-S.	ECT reduces systemic inflammation but improvements do not directly correlate with symptom reduction; suggests multiple independent therapeutic mechanisms.
Ali et al., 2024 (28)	72 BD patients (median age 30; 55.6% male)	Prospective cohort study	Bitemporal brief-pulse ECT, 3 sessions/week; total of 4 sessions over 10 days	RDW, PDW (hematological inflammatory markers)	Post-ECT: PDW ↑ (platelet activation), RDW ↓ (reduced systemic inflammation)	Clinical improvement observed; No significant differences in platelet count, mean platelet volume, or plateletcrit.	ECT appears to modulate systemic inflammation via hematological pathways, particularly platelet-related immune activity; findings suggest potential for immune-targeted adjunctive therapies in BD.
Hough et al., 2024 (29)	40 TRD patients (mean age 41.78; 55% female); 26 healthy controls (mean age 40.50; 65% female)	Longitudinal observational study	Predominantly right unilateral ultrabrief pulse ECT (5x seizure threshold), switched to bilateral if needed; mean 11.5 sessions (range 6–22)	CRP, IL-6, TNF-α	Biphasic inflammatory trajectory post-ECT: IL-6 and CRP ↑ (T1→T2); IL-6 ↓ to baseline levels at T3, CRP remained ↑ at T3.	IL-6 early elevations (T2) were linked to greater affective/cognitive improvement if IL-6 remained high at T3; the opposite was observed in those with lower IL-6 at T3. Similar trend seen with CRP and neurovegetative symptoms.	ECT induces an early immune activation phase followed by partial resolution. Persistently elevated CRP post-treatment suggests that long-term immune modulation remains incomplete, highlighting the need for adjunctive anti-inflammatory approaches.
Gaarden et al., 2025 (30)	64 elderly TR-MDD patients (mean age 75.2; 54.7% female); 18 non-depressed controls (mean age 78.1; 66.7% female)	Longitudinal exploratory study	Bilateral/unilateral ECT, avg. 9 sessions, 2 per week	27 immune markers (TNF, IFN-γ, PDGF-BB, IL-6, etc.)	Post-ECT: 19 immune markers ↓; 23 markers remained ↑ in patients vs. controls at follow-up.	47% remission rate (HDRS-17 <8); no significant difference in inflammation between responders and non-responders.	Persistent pro-inflammatory trait in elderly post-ECT suggests chronic low-grade inflammation in TR-MDD patients.

ECT, Electroconvulsive Therapy; MDD, Major Depressive Disorder; TRD, Treatment-Resistant Depression; TR-MDD, Treatment-Resistant Major Depressive Disorder; LLD, Late-Life Depression; BD, Bipolar Disorder;

HDRS, Hamilton Depression Rating Scale; MADRS, Montgomery-Åsberg Depression Rating Scale; BPRS, Brief Psychiatric Rating Scale; CGI-S, Clinical Global Impression, Severity Scale; IL-1β, Interleukin-1 Beta; IL-2R, Interleukin-2 Receptor; IL-4, Interleukin-4; IL-5, Interleukin-5; IL-6, Interleukin-6; IL-8, Interleukin-8; IL-10, Interleukin-10; IL-17, Interleukin-17; IL-18, Interleukin-18; TNF-α, Tumor Necrosis Factor Alpha; CRP, C-Reactive Protein; CRP-albumin ratio, C-Reactive Protein to Albumin Ratio; CAR, Cortisol-to-Albumin Ratio; NLR, Neutrophil-to-Lymphocyte Ratio; MLR, Monocyte-to-Lymphocyte Ratio; PLR, Platelet-to-Lymphocyte Ratio; RDW, Red Cell Distribution Width; PDW, Platelet Distribution Width; SII, Systemic Immune-Inflammation Index; NF-κB, Nuclear Factor Kappa B; NLRP3, NLR Family Pyrin Domain Containing 3 Inflammasome; RANTES, Regulated on Activation, Normal T Cell Expressed and Secreted; IP-10, Interferon Gamma-Induced Protein 10; MIP-1α, Macrophage Inflammatory Protein 1-alpha; IFN-γ, Interferon Gamma; PDGF-BB, Platelet-Derived Growth Factor Subunit B; HMGB1, High Mobility Group Box-1 Protein; sRAGE, Soluble Receptor for Advanced Glycation End Products; BDNF, Brain-Derived Neurotrophic Factor; CSF, Cerebrospinal Fluid; CNS, Central Nervous System; WMS-R, Wechsler Memory Scale–Revised; MMSE, Mini-Mental State Examination.

↑ = increased levels or expression; ↓ = decreased levels or expression.

**Table 2:** Main features of studies evaluating neuroplasticity, neuroimmune processes, and brain changes related to ECT in mood disorders.

Study, year	Sample	Study Design	ECT Protocol	Biomarkers Assessed	Key Findings	Clinical Outcomes	Conclusions & Implications
Han et al., 2023 (31)	204 severe MDD patients (102 ECT-treated: mean age 46.3; 56.86% male 102 non-ECT: mean age 45.8; 58.82% male)	Prospective cohort study	Bilateral ECT, 3 sessions/week, up to 12 sessions	IL-6, BDNF, Cortisol	In ECT patients post-treatment: IL-6 ↑ (less than in non-ECT group); BDNF ↓ in both groups but better preserved in ECT; Cortisol unchanged.	ECT group showed greater depression symptom relief, improved QoL and cognition, with 68% achieving remission vs. 42% in the non-ECT group.	ECT may attenuate excessive inflammatory responses in MDD but does not fully normalize IL-6 levels; findings support ECT’s role in neuroplasticity; relative BDNF preservation post-ECT.
Xu et al., 2023 (32)	40 TRD patients (mean age 22.2; 60% female); 35 healthy controls (mean age 23.1; 65.7% female)	Prospective cohort study	Modified bifrontal ECT, Thymatron IV; 3 sessions/week until remission (HAMD ≤7); mean duration 14.6 ± 5.8 days	GFAP, S100β, CD81, IL-1β, IL-4, IL-6, IL-10, IL-17A, TNF-α, IFN-γ	Post-ECT: significant ↓ in GFAP, S100β, CD81, IFN-γ, and IL-4; suggesting astrocytic deactivation and immune modulation. IL-4 ↓ correlated with MADRS vegetative symptom improvement.	Significant reduction in MADRS total score, with improvement across all symptom subscales; higher baseline astrocyte/inflammatory markers correlated with greater symptom severity and cognitive dysfunction.	ECT reduces astrocyte activation and neuroinflammation, potentially contributing to therapeutic efficacy in TRD; findings support targeting glial and immune mechanisms in future interventions.
Abe et al., 2023 (33)	25 MDD patients (median age 54.2; 56% female); 25 healthy controls (median age 53.2; 56% female)	Prospective cohort study	Bilateral ECT, Thymatron System IV, avg. 10.5 sessions	HMGB1, sRAGE	HMGB1 and sRAGE: no difference at baseline (MDD vs. controls); no post-ECT change in either marker.	Significant reduction in HDRS scores post-ECT; No correlation between inflammatory markers and symptom improvement.	Findings challenge the hypothesis that HMGB1 and sRAGE play a role in the inflammatory response to ECT. Suggests a more complex relationship between these markers and depression pathophysiology.
Brooks et al., 2024 (34)	20 TRD patients (mean age 42.5; 50% female)	Longitudinal observational study	Predominantly right unilateral ultra-brief pulse ECT (6x seizure threshold); switched to bilateral brief-pulse (2x ST) if no response after 10 sessions; mean 11.1 sessions (range 6–16), 3 sessions/week	L-6, IL-8, TNF-α, MRI (voxel-based morphometry)	Post-ECT IL-8 ↓ correlated with symptom improvement; GM volume ↓ in 4 clusters (incl. right insula & Brodmann’s area 22), one associated with depression severity at 6 months.	No direct mediation between inflammatory changes and neuroimaging structural alterations.	Structural changes post-ECT may represent neuroadaptive processes rather than neurotoxicity; highlights the need for long-term studies on brain plasticity in ECT-treated TRD patients.
Andreou et al., 2022 (35)	42 patients with TRD (mean age: 43.15; 54.8% female)	Longitudinal cohort study	Right unilateral ultrabrief pulse ECT, 3 sessions/week; ~30% transitioned to bilateral; average 10.88 sessions	Serum IL-6, IL-8, TNF-α, CRP; diffusion-weighted DWI MRI assessing free-water corrected fractional anisotropy (FAt) in 17 white matter tracts	Baseline: IL-8 ↑ in patients vs. controls; Post-ECT: IL-8 change correlated with FAt changes in right cingulum and SLF (responders only).	Only responders showed neuroimmune coupling between IL-8 and FAt in the right cingulum and SLF; absent in non-responders.	ECT may promote glia-mediated white matter remodeling via IL-8 signaling in responders. Findings suggest neuroimmune modulation as a mechanistic component of ECT in TRD.
Loef et al., 2023 (36)	12 ECT-treated depressed patients (mean age 54.25); 10 non-ECT depressed patients (mean age 66.9); 15 healthy controls (mean age 68.87)	Post-mortem histopathological study	ECT-treated patients received right unilateral and/or bilateral ECT within 5 years prior to death. Detailed parameters (number of sessions, frequency, stimulus intensity) not reported.	DCX, STMN1 (neuroplasticity markers), GFAP, Iba1 (astrocytic/microglial markers)	Post-ECT: ↑ DCX+ cells in CA4 (vs. controls) & SGZ (vs. non-ECT & controls); ↑ STMN1+ in SGZ (ECT group); no microglial/astrocytic activation or cytoarchitectural damage.	Neuroplasticity markers suggest an ECT-induced increase in neuronal differentiation, which may contribute to long-term symptom improvement in mood disorders.	ECT enhances hippocampal neurogenesis without triggering chronic inflammation or gliosis, reinforcing its effect in neuroplasticity and potentially sustaining its antidepressant effects.

ECT – Electroconvulsive Therapy; MDD – Major Depressive Disorder; TRD – Treatment-Resistant Depression; QoL – Quality of Life; HDRS – Hamilton Depression Rating Scale; MADRS – Montgomery-Åsberg Depression Rating Scale; IL-1β – Interleukin-1 Beta; IL-4 – Interleukin-4; IL-6 – Interleukin-6; IL-8 – Interleukin-8; IL-10 – Interleukin-10; IL-17A – Interleukin-17A; TNF-α – Tumor Necrosis Factor Alpha; IFN-γ – Interferon Gamma; CRP – C-Reactive Protein; BDNF – Brain-Derived Neurotrophic Factor; GFAP – Glial Fibrillary Acidic Protein; S100β – S100 Calcium-Binding Protein B; CD81 – Cluster of Differentiation 81; STMN1 – Stathmin 1; DCX – Doublecortin; Iba1 – Ionized Calcium-Binding Adapter Molecule 1; MRI – Magnetic Resonance Imaging; DWI – Diffusion-Weighted Imaging; FAt – Free-Water Corrected Fractional Anisotropy; GM – Gray Matter; SLF – Superior Longitudinal Fasciculus; SGZ – Subgranular Zone; CA4 – Cornu Ammonis Area 4 (hippocampal subregion).

↑ = increased levels or expression; ↓ = decreased levels or expression.

**Table 3:** Main features of studies on genetic, epigenetic, microRNA, and mitochondrial biomarkers after ECT in mood disorders.

Study, year	Sample	Study Design	ECT Protocol	Biomarkers Assessed	Key Findings	Clinical Outcomes	Conclusions & Implications
Carvalho Silva et al., 2024 (37)	32 TRD patients (mean age 56.9; 68.7% female)	Prospective longitudinal study	Bilateral brief-pulse ECT (Thymatron DG), 3 sessions/week; mean 7.4 ± 2.2 sessions; completed based on clinical judgment	DNA methylation changes, epigenetic markers	54 DMRs and 3 DMPs identified post-ECT; none remained significant after FDR correction.	No direct correlation between epigenetic changes and symptom severity.	ECT modulates epigenetic markers; however, findings did not reach statistical significance after correction; results highlight the need for further studies on epigenetic biomarkers predicting treatment response.
Moschny et al., 2020 (38)	87 patients with TRD (59 in cross-sectional, 28 in longitudinal cohort; mean age 51.9; 51.7% female)	Prospective observational study	Right unilateral ECT (3 sessions/week, up to 4 weeks); bilateral ECT considered after 2 weeks if non-response	DNA methylation of *PLAT* (t-PA) and *SERPINE1* (PAI-1) in B cells, T cells, monocytes, NK cells, and whole blood	No differences in t-PA or PAI-1 methylation between ECT remitters and non-remitters (baseline and post-ECT); significant variation in t-PA methylation across immune cell types, but stable across ECT course.	33 patients achieved remission; methylation profiles did not correlate with clinical improvement.	Epigenetic signatures of *t-PA* and *PAI-1* are not predictive of ECT response. Findings highlight the importance of analyzing DNA methylation in specific immune cell subtypes rather than whole blood samples.
Maier et al., 2023 (39)	31 patients with MDD (mean age: 55; 64.5% female); 19 unmedicated depressed controls; 20 healthy controls	Prospective observational study	Right unilateral ECT, 3 sessions/week, average of 10 ± 4 sessions; Thymatron IV system	DNA methylation of NR3C1 (glucocorticoid receptor) and POMC in PBMCs	Baseline: NR3C1 methylation ↑ in non-responders; Post-ECT: NR3C1 methylation ↓ in both responders and nonresponders; POMC methylation ↓ in responders but ↑ in nonresponders.	NR3C1 methylation was higher in non-responders at baseline but decreased in both groups post-ECT. POMC methylation decreased in responders and increased in non-responders, indicating an epigenetic signature associated with response.	ECT induces short-term epigenetic changes in HPA-axis-related genes. NR3C1 methylation may act as a baseline biomarker of treatment response; POMC methylation changes may reflect stress regulation dynamics associated with ECT outcome.
Kaurani et al., 2023 (40)	64 TRD patients (mean age 55.9; 59.38% female)	SmallRNA sequencing analysis	Bilateral ECT (placement adjusted as needed), avg. 12 sessions; titration based on seizure threshold or age	miR-223-3p, IL-6, IL-1β, TNF-α, NLRP3	Baseline: miR-223-3p ↓ and IL-6, TNF-α ↑ in responders; Post-ECT: miR-223-3p ↑, IL-6 and TNF-α ↓ in responders; miR-223-3p discriminated responders from non-responders.	Significant symptoms improvement (≥50% MADRS decrease) in 62.5% patients; miR-223-3p down-regulated at baseline among responders; higher post-ECT miR-223-3p expression associated with greater depressive symptom improvement.	miR-223-3p may serve as a biomarker for ECT response, linking immune-inflammatory pathways to antidepressant effects. Findings highlight the potential for miRNA-based diagnostics in TRD treatment and further research on inflammatory modulation.
Galbiati et al., 2025 (41)	27 TRD patients (mean age 58.1; 67% female	Prospective longitudinal study	Bilateral brief-pulse ECT (Thymatron DG); 3 sessions/week; mean 7.9 ± 2.7 sessions; dosing based on age or seizure threshold titration	miR-30c-5p, miR-324-3p, miR-95-3p, miR-194-5p, miR-195-5p, miR-19b-3p, let-7i-5p, miR-497-5p	Downregulation of 8 miRNAs post-ECT; symptom improvement correlated with changes in miR-30c-5p and miR-324-3p in responders; Only miR-324-3p correlated with symptom relief in the whole sample.	Significant symptoms improvement reduction in MADRS scores; 18 out of 27 classified as responders (≥50% symptom reduction).	Findings suggest that miR-30c-5p and miR-324-3p may be implicated in the antidepressant mechanism of ECT and could serve as potential biomarkers for treatment response.
Ryan et al., 2023 (42)	100 MDD patients (mean age 56.36; 62% female) treated with ECT; 89 healthy controls (mean age 53.42; 66.29% female)	Observational cohort study	Bilateral ECT (bitemporal at 1.5× ST or unilateral at 6× ST); mean 8.03 ± 2.51 sessions	mtDNAcn, telomere length, inflammatory markers (IL-6, IL-10, TNF-α, and CRP)	Baseline: mtDNAcn ↑ in MDD vs. controls; no correlation with inflammation, telomere length, or depression severity; mtDNAcn not predictive of ECT response/remission.	No association between mtDNAcn levels and ECT response or remission.	Findings suggest mitochondrial dysfunction in depression; however, mtDNAcn did not serve as a predictor for ECT treatment outcomes. Further research is warranted on mitochondrial pathways in depression.

ECT, Electroconvulsive Therapy; MDD, Major Depressive Disorder; TRD, Treatment-Resistant Depression; MADRS, Montgomery-Åsberg Depression Rating Scale; IL-1β, Interleukin-1 Beta; IL-6, Interleukin-6; IL-10, Interleukin-10; TNF-α, Tumor Necrosis Factor Alpha; CRP, C-Reactive Protein; NLRP3, NLR Family Pyrin Domain Containing 3 Inflammasome; miRNAs, microRNAs; miR-223-3p, microRNA 223-3p; miR-30c-5p, miR-324-3p, miR-95-3p, miR-194-5p, miR-195-5p, miR-19b-3p, let-7i-5p, miR-497-5p, Specific microRNAs studied; mtDNAcn, Mitochondrial DNA Copy Number; DMRs, Differentially Methylated Regions; DMPs, Differentially Methylated Positions; FDR, False Discovery Rate; NR3C1, Nuclear Receptor Subfamily 3 Group C Member 1 (Glucocorticoid Receptor Gene); POMC, Proopiomelanocortin; PLAT, Tissue-Type Plasminogen Activator Gene; SERPINE1, Plasminogen Activator Inhibitor-1 Gene; PBMCs, Peripheral Blood Mononuclear Cells; ST, Seizure Threshold.↑ = increased levels or expression; ↓ = decreased levels or expression.

## Results

A total of 26 studies published between 2020 and 2025 were included, investigating the effects of ECT on immune, inflammatory, and cellular processes in patients with mood disorders (e.g., MDD and BD). Sample sizes varied widely, ranging from small pilot studies to large cohorts. The methodologies included longitudinal biomarker investigations, neuroimaging, molecular analyses, post-mortem histopathology, DNA/small RNA sequencing, and mtDNAcn assessments.

### Inflammatory markers and antidepressant response

Studies focused on the impact of ECT on peripheral inflammatory markers, reporting reductions in systemic inflammation following treatment. In a prospective cohort study, Du et al. (2024) analyzed inflammatory biomarkers and depressive symptoms in 38 adolescents with severe MDD (median age [interquartile range]: 15 [14–16]; 81.6% female) compared to 29 healthy-controls (median age [interquartile range]: 15 [14.5–16.5]; 53.8% female). Baseline blood samples (e.g., interleukin-1 beta [IL-1β], interleukin-6 [IL-6], and interleukin-10 [IL-10]) were collected before treatment and after completing 6–8 ECT sessions spanning two weeks. The outcomes showed significant post-ECT decreases in IL-1β and IL-6, and an increase in IL-10. Interestingly, improvements in depressive symptoms (as assessed by the 17-item Hamilton Depression Rating Scale scores [HDRS]) were correlated with reductions in IL-1β (r = -0.343, p = 0.035) and in IL-6 (r = -0.403, p = 0.012), suggesting a link between anti-inflammatory and antidepressant effects of ECT in this population (17).

Mindt et al. (2020) examined CSF and plasma levels of 25 cytokines in 12 TRD patients (mean ± SD age: 59 ± 21.9; 58.3% female) undergoing ECT, focusing on central immune modulation. CSF levels of IL-6 and IL-1β showed negative correlations with the number of ECT sessions, suggesting progressive reductions over the treatment course. Moreover, patients who achieved remission exhibited reductions in CSF IL-17, MIP-1α, RANTES, and IL-2R, while IP-10 levels increased to a lesser extent compared to non-remitters. Such findings indicate that ECT may modulate central immune processes beyond peripheral inflammation (18). In addition, Kruse et al. (2020) analyzed serum levels of IL-6 and IL-8 in 40 patients with major depression (mean age ± SD: 41.8 ± 13.7; 55% female) treated with ECT. Their findings revealed sex-specific differences in inflammatory dynamics, with increases in IL-8 associated with symptom improvement in women but not in men. These results suggest that IL-8 may serve as a sex-dependent biomarker for ECT response and support further investigations of gender-specific immune signatures (19). In contrast, Ryan et al. (2022a) found no significant post-treatment changes in a panel of classic inflammatory cytokines. The authors examined plasma concentrations of CRP, IL-6, IL-10, TNF-α, and IL-1β in 86 patients with MDD (mean age ± SD: 55.1 ± 14.65; 61.63% female) and 57 healthy controls (mean age ± SD: 51 ± 12.7; 66.1% male). Although IL-6 and TNF-α were elevated at baseline in depressed individuals, these levels remained unchanged after ECT; no associations were found between cytokine concentrations and clinical or cognitive outcomes. IL-1β was undetectable in most samples. Taken together with other studies, these results illustrate the heterogeneity in ECT’s effects on systemic inflammatory markers and depressive symptoms (20).

### Inflammation and treatment response in late-life depression

Studies have specifically investigated the immunoinflammatory response to ECT in older adults, aiming to clarify its relationship with treatment efficacy in late-life depression (LLD). Carlier et al. (2021) conducted a prospective cohort study evaluating the inflammatory response to ECT in 99 older adults with depression (mean age ± SD: 72.8 ± 8.3; 66.7% female). Patients received right unilateral ECT with dose titration; non-responders were switched to bilateral ECT after six sessions. Across a median of 11 sessions, small reductions in CRP and IL-6 were observed, although these changes did not reach statistical significance after Bonferroni correction. IL-10 and TNF-α levels remained unchanged. Notably, higher baseline CRP were significantly higher among remitters. Clinically, 77.8% of patients responded (e.g., ≥50% MADRS score reduction), and 66.7% achieved full remission (MADRS < 10) (21).

In a complementary analysis of the same cohort, Loef et al. (2021) investigated how the balance between inflammatory activity and neuroplastic support might influence ECT outcomes. In addition to assessing serum IL-6, TNF-α, and BDNF levels, the authors calculated the IL-6/BDNF and TNF-α/BDNF ratios to represent the relative dominance of immune versus neuroplastic signaling. While no direct associations were found between individual biomarker levels and MADRS scores at any time point, a significant interaction effect was observed: the relationship between BDNF and depressive severity became more negative in the presence of elevated TNF-α levels (p = 0.020). Moreover, a higher TNF-α/BDNF ratio was significantly associated with greater symptomatic burden (p = 0.007), suggesting that imbalance skewed toward proinflammatory signaling may hinder neuroplastic recovery and ECT effectiveness. Thus, these combined results suggest that ECT outcomes in LLD may be influenced by post-treatment biomarker changes, pre-existing inflammatory burden, and interactions with neuroplastic features (22).

### Inflammatory dynamics and cognitive adverse events

Other studies investigated the potential relationship between inflammatory responses and cognitive adverse events of ECT in depression treatment. Tian et al., 2021 conducted a prospective controlled study including 40 patients with first-episode, drug-naive MDD (mean age ± SD: 49.5 ± 7.32; 50% female) and 40 healthy controls matched for age, sex, and education (mean age ± SD: 48.3 ± 6.76; 55% female). Individuals underwent 12 sessions of modified ECT (three per week), initiated alongside a stable antidepressant regimen. Blood samples were collected before and after treatment to assess peripheral levels of NLRP3 inflammasome, interleukin-18 (IL-18), and nuclear factor kappa B (NF-κB). Results showed significant post-ECT increases in all three markers (NLRP3: p < 0.01; IL-18: p < 0.05; NF-κB: p < 0.01). Notably, 70% of the patients experienced cognitive impairment, defined as a score below 50 on the Wechsler Memory Scale–Revised. The elevation of inflammatory markers was significantly associated with lower cognitive scores, suggesting that inflammatory activation, particularly via NLRP3-related pathways, may contribute to cognitive adverse events observed in a subset of persons undergoing ECT (23).

Ryan et al. (2022b) further assessed 62 patients with depression (mean age ± SD: 56.84 ± 15.3; 66.1% female) undergoing bilateral ECT and analyzed neutrophil-to-lymphocyte ratio (NLR), platelet-to-lymphocyte ratio (PLR), monocyte-to-lymphocyte ratio (MLR), and systemic immune-inflammation index (SII). While elevated PLR and SII correlated with lower HAMD-24 scores at baseline, higher NLR, PLR, and SII values were significantly associated with prolonged time to reorientation following ECT (24). Focusing on psychomotor aspects, Belge et al. (2021) investigated the relationship between IL-6 and depressive symptoms in a cohort of 62 participants undergoing ECT (mean age ± SD: 58.2 ± 14.8; 77.4% female). Following 8 to 12 bilateral sessions, serum IL-6 levels showed a modest yet statistically significant reduction (median from 0.74 to 0.65 pg/mL; p = 0.02). Notably, IL-6 changes correlated specifically with improvements in psychomotor retardation, as measured by a validated scale. Depressive symptoms also improved substantially, with HDRS-17 scores decreasing from 24.6 to 8.7 (p < 0.0001) (25).

Additionally, Carlier et al. (2021) examined whether baseline inflammatory markers were associated with cognitive outcomes in a cohort of 97 older adults with unipolar depression undergoing ECT (mean age ± SD: 73.1 ± 8.1; 67% female). Serum CRP, IL-6, IL-10, and TNF-α levels were measured prior to treatment, and Mini-Mental State Examination (MMSE) scores were assessed before, weekly during, and immediately after ECT. Higher baseline TNF-α and IL-10 were significantly associated with lower MMSE scores during treatment, while elevated baseline CRP predicted poorer cognitive performance immediately after ECT. These findings suggest that pre-existing inflammation may predispose older individuals to greater cognitive vulnerability during ECT (26).

Collectively, these studies indicate that peripheral inflammation may correlate with cognitive adverse events of ECT, particularly in older adults. While some markers reflect symptom improvement, others—such as TNF-α, IL-10, and inflammatory ratios—may signal greater cognitive vulnerability.

### Hematological and systemic immune indices

In 2025, Kirlioglu Balcioglu et al. published a retrospective cohort study investigating the effects of ECT on inflammatory markers in a sample of 156 patients (mean ± SD age: 36.09 ± 13.99; 58.3% male) diagnosed with psychotic and mood disorders. Their results showed significant post-ECT decreases in CRP, CRP–albumin ratio, NLR, MLR, PLR, and cortisol-to-albumin ratio (CAR). Symptom improvement was also observed, with a reduction in the Brief Psychiatric Rating Scale (BPRS) score from 59.93 ± 10.95 pre-ECT to 21.98 ± 5.71 post-ECT (p < 0.01) and a Clinical Global Impression – Severity (CGI-S) score decrease from 6.04 ± 0.68 to 1.23 ± 0.52 (p < 0.01) (27).

Similarly, Ali et al. (2024) studied 72 BD patients (median age [range]: 30 [17-57]; 55.6% male) and found post-ECT shifts in complete blood count-derived inflammatory markers, such as platelet distribution width (PDW), which increased from 11.7 ± 2.2 pre-ECT to 12.7 ± 2.6 post-ECT (p = 0.004), and red cell distribution width (RDW), which decreased from 14.1 ± 1.1 to 13.6 ± 1.0 (p < 0.001). No significant differences were found in platelet count, mean platelet volume, and plateletcrit, showing that ECT did not impair platelet homeostasis despite the other observed hematological changes (28). Ryan et al. (2022b), as previously discussed, also explored whether pre-treatment hematological markers—specifically blood cell-derived ratios such as NLR, PLR, MLR, and the SII—could predict cognitive and mood outcomes following ECT. While no associations with mood improvement were observed, higher baseline values of NLR, PLR, and SII were significantly associated with delayed recovery of orientation (24).

### Biphasic inflammatory responses and long-term immune activity

Some studies showed a biphasic trajectory of systemic inflammation following ECT. Hough et al. (2024) examined the levels of peripheral inflammatory markers in 40 patients with treatment-resistant depression (TRD) (mean ± SD age: 41.78 ± 13.73; 55% female) undergoing ECT, compared to 26 healthy controls (mean ± SD age: 40.50 ± 13.42; 65% female). Plasma inflammatory markers were evaluated at three time points: (T1) pre-treatment, (T2) post-second ECT session, and (T3) post-treatment. The study found a transient increase in CRP and IL-6 levels from T1 to T2, followed by a decline from T2 to T3. While IL-6 returned to baseline levels post-treatment, CRP remained elevated compared to pre-treatment values. Notably, no direct correlation was identified between general inflammatory changes and symptom improvement; however, a dynamic interaction between the early/acute inflammatory response and post-treatment inflammation compared to baseline correlated with clinical outcomes. While there was no direct correlation between inflammatory changes and overall symptom reduction, larger early elevations in IL-6 at T2 were associated with more remarkable improvement in affective and cognitive symptoms among individuals with higher levels of IL-6 post-treatment (T3), while a trend to the opposite was observed in those with lower IL-6 levels after the intervention. A similar trend was observed between CRP levels trajectory and neurovegetative symptoms (29).

In 2025, Gaarden et al. conducted a longitudinal study including 64 elderly patients with treatment-resistant unipolar depression (mean ± SD age: 75.2 ± 6.3; 54.7% female) treated with ECT and 18 non-depressed controls (mean ± SD age: 78.1 ± 4.8; 66.7% female). Blood samples for analysis of 27 immune markers were collected from patients pre, mid, and post-treatment and at 12 weeks of follow-up; for controls, the markers were assessed through identical methods at baseline and at 8 weeks of follow-up. The analysis showed that 47% achieved clinical remission (as classified by a score < 8 on the 17-item HDRS). Although a significant decrease of 19 immune markers was observed from pre- to post-treatment in the patients’ intra-group comparisons, higher concentrations of 23 immune markers were observed among patients compared to controls at the follow-up. Moreover, there were no differences in immune markers concentrations between responder and non-responder patients. These results raised questions about whether prolonged inflammatory activity in older populations might influence long-term treatment efficacy or relapse risk (30).

The main characteristics of the studies evaluating peripheral inflammatory and hematological biomarkers in relation to ECT outcomes are shown in **Table 1**.

### Neuroplasticity, neuroimmune processes, and post-mortem findings

Beyond the ECT’s impact on inflammation, Han et al., in 2023, also added evidence of neuroplasticity in a prospective cohort study including severe MDD patients divided into 102 ECT-treated (mean ± SD age: 46.3 ± 11.2; 56.86% male) and 102 non-ECT treated (mean ± SD age: 45.8 ± 10.9; 58.82% male). The primary outcome was the change in HDRS scores from baseline to 12 weeks. Both groups showed significant symptom improvement, whereas the reduction in HDRS scores was significantly greater in the ECT group (-19.6 ± 6.4) compared to the non-ECT group (-14.2 ± 7.2, p < 0.001). Moreover, 68% of the ECT-treated patients achieved remission (HDRS ≤ 7) compared to 42% in the non-ECT group. Biomarker analysis revealed that BDNF levels decreased in both groups; however, the decline was significantly attenuated in the ECT group (-12.8 ± 4.6 vs. -18.6 ± 5.8, p < 0.001), suggesting a neuroprotective effect. Similarly, IL-6 increased in both groups, although with a less pronounced rise in the ECT group (+2.2 ± 0.9 vs. +3.4 ± 1.2, p < 0.001). No significant differences were found in cortisol levels between the groups. In addition, quality of life (as assessed by the WHO Quality of Life Brief Version) and cognitive function (evaluated by the Montreal Cognitive Assessment) improved more significantly in the ECT group. The findings of this study suggested that ECT led to significant symptomatic improvement and may influence neuroplasticity and inflammation in severe MDD (31).

Several studies explored neuroimmune interactions and cellular changes associated with ECT. Xu et al. (2023) investigated astrocytic markers and inflammatory cytokines in 40 TRD patients (mean ± SD age: 22.2 ± 4.5; 60% female) undergoing ECT and 35 healthy controls (mean ± SD age: 23.1 ± 3.9; 65.7% female). Pre-ECT, levels of glial fibrillary acidic protein (GFAP), S100 calcium-binding protein B (S100β), CD81, IL-1β, IL-4, IL-6, IL-10, IL-17, tumor necrosis factor-alpha (TNF-α), and inflammatory markers interferon γ (IFN-γ) were significantly higher in TRD patients than in controls. These elevations were positively correlated with cognitive dysfunction (r = 0.62, p < 0.001). Following ECT, there were significant decreases of GFAP, S100β, CD81, as well as IL-4 and IFN-γ in the TRD group compared to controls. Alongside these changes, patients showed significant improvement in all subscales of the Montgomery-Åsberg Depression Rating Scale (MADRS). Furthermore, the IL-4 decrease correlated with reductions in the MADRS vegetative subscale scores. This study added that ECT may also exert anti-inflammatory effects through astrocytic activity modulation in depressed individuals (32).

Abe et al. (2023) investigated the role of high mobility group box-1 protein (HMGB1) and soluble receptor for advanced glycation end-products (sRAGE) in the context of ECT, analyzing 25 MDD patients (median age [range]: 54.2 [48–63]; 56% female) and 25 healthy controls (median age [range]: 53.2 [47–61]; 56% female). HMGB1 is a damage-associated molecular pattern protein involved in neuroinflammation, blood-brain barrier dysfunction, and immune activation, while sRAGE functions as a decoy receptor that neutralizes pro-inflammatory HMGB1 signaling. Given prior evidence linking elevated HMGB1 to depression and stress-related neuroimmune dysregulation, it was hypothesized that ECT might exert therapeutic effects by downregulating HMGB1-mediated inflammatory pathways or modulating sRAGE expression. Surprisingly, however, no significant differences in HMGB1 or sRAGE levels at baseline were found between MDD patients and healthy controls. While ECT led to symptoms improvement (as assessed by the 21-item HDRS), it did not induce any significant post-treatment changes (HMGB1: p = 0.677; sRAGE: p = 0.922) (33).

A study by Brooks et al. (2024) assessed inflammation patterns, neuroimaging data, and its correlations with depressive symptoms in 20 TRD patients (mean ± SD age: 42.5 ± 13.7; 50% female) undergoing ECT. Magnetic resonance imaging (MRI) and inflammation markers (e.g., IL-6, IL-8, and TNF- α) from blood samples were obtained after an index ECT and six months after. MRI was evaluated with voxel-based morphometry. The results showed a correlation between IL-8 decrease and depression symptoms improvement, as evaluated by the 17-item HDRS (r = 0.65; p = 0.01). However, despite this correlation, inflammatory changes did not directly mediate neuroimaging structural changes and depressive symptoms. Four clusters of gray matter volume significant reductions were found; one cluster, including Brodmann’s area 22 and right insula, correlated with higher severity of symptoms in HAM-D over six months. The author discussed that such structural changes may not necessarily be a sign of neurotoxicity but part of a dynamic neuroadaptive process (34).

In another study exploring possible correlations between neuroinflammation and structural brain changes following neuromodulation, Andreou et al. (2022) investigated 42 TRD patients (mean age ± SD: 43.15 ± 13.82; 54.8% female) undergoing ECT (right unilateral ultra-brief pulse stimulus; 3 sessions/week; ~30% transitioned to bilateral). Pre- and post-treatment serum levels of IL-6, IL-8, TNF-α, and CRP were measured alongside diffusion-weighted MRI to assess free-water corrected fractional anisotropy (FAt) in 17 white matter tracts. Among responders (≥50% MADRS reduction), changes in IL-8 significantly correlated with FAt alterations in the right cingulum and superior longitudinal fasciculus (r = 0.65–0.70), suggesting a role for IL-8 in glia-mediated white matter remodeling. These neuroimmune associations were absent in non-responders, reinforcing the hypothesis that inflammatory signaling may support therapeutic plasticity induced by ECT (35).

Furthermore, post-mortem histopathological analyses contributed to elucidating the neurobiological effects of ECT. In 2023, Loef et al. published a first explorative study assessing direct histopathological evidence of ECT-induced neuroplasticity in human post-mortem brain tissue, examining hippocampal samples from 12 patients with bipolar or unipolar depression (mean ± SD age: 54.25 ± 21.85; 58.3% male) who had received ECT within the five years preceding death, compared to 10 depressed donor patients who were not treated with ECT (mean ± SD age: 66.9 ± 13.47; 60% female), and 15 healthy control donors (mean ± SD age: 68.87 ± 16.02; 53.3% female). In subjects previously treated with ECT, the analyses showed a significantly higher proportion of cells positive for doublecortin (DCX) in the hippocampal CA4 area (compared to healthy control donors) and subgranular zone (compared to both non-ECT depressed patients and healthy controls donors). DCX is a marker for the presence of young neurons and cellular plasticity. In addition, a higher percentage of positive stathmin 1 cell (STMN1), another marker for neuroplasticity, was found in the hippocampal subgranular zone of ECT-treated subjects compared with non-ECT depressed patients and healthy control donors. Notably, there were no evident differences in inflammatory markers reflecting microglial or astrocytic activity, as well as in structural changes (e.g., major hippocampal cell loss, overt cytoarchitectural changes) among the three groups. Based on these results, the authors discussed that ECT may enhance hippocampal neurogenesis without promoting chronic inflammatory responses or gliosis (36).

The main characteristics of studies investigating neuroplastic and structural brain changes in relation to ECT in mood disorders are shown in **Table 2**.

### Molecular, epigenetic, and mitochondrial function modifications

Adding novelty to the field, molecular and epigenetic analyses investigated ECT-induced changes. In 2024, Carvalho Silva et al. published an epigenome-wide longitudinal study investigating DNA methylation in 32 TRD patients (mean ± SD age: 56.9 ± 14.3; 68.7% female) undergoing ECT. Clinical severity (evaluated with the MADRS) and ECT outcomes were assessed at baseline (T0) and 1 month after intervention (T1); genome-wide methylation was analyzed at T0 and T1. Analyses showed three differentially methylated probes (DMPs), with two annotated in genes CYB5B and PVRL4; nonetheless, these probes were not significant after false discovery rate (FDR) correction. In a covariate analysis including changes in clinical symptoms (as assessed by the MADRS), four DMPs were found annotated in the genes FAM20C, EPB41, OTUB1, and ADARBI1. Considering response status as a covariate to the model, three DMPs were detected, with two annotated in the genes IQCE and FAM20C. However, none of these probes remained significant after the FDR correction. Similarly, fifty-four differentially methylated regions (DMRs) were found, alongside the identification of 21 DMRs for symptoms variations and 26 DMRs for response status. Again, none of these regions remained significant after the FDR correction (37). In contrast to genome-wide approach, Moschny et al. (2020) analyzed DNA methylation patterns of tissue-type plasminogen activator (t-PA; gene: PLAT) and its inhibitor PAI-1 (SERPINE1) in sorted immune cells (B cells, T cells, monocytes, and NK cells), as well as in whole blood, from 87 TRD participants undergoing ECT (mean ± SD age: 51.9 ± 16.6; 51.7% female). Although significant variability in methylation across immune cell types was observed, no consistent differences in PLAT or SERPINE1 methylation were found between remitters and non-remitters. Clinical response (≥50% MADRS reduction) was achieved in 33 patients; however, methylation profiles were not associated with symptom improvement (38).

Maier et al. (2023) conducted a prospective observational study to examine the effects of ECT on DNA methylation in genes related to hypothalamic–pituitary–adrenal (HPA) axis regulation. The study included 31 patients with MDD (mean ± SD age: 55 ± 16; 64.5% female), alongside 19 unmedicated depressed controls and 20 healthy controls. All patients underwent right unilateral ECT (3 sessions/week; average of 10 ± 4 sessions) using the Thymatron IV system. Peripheral blood mononuclear cells (PBMCs) were collected to assess methylation patterns of NR3C1 (encoding the glucocorticoid receptor) and POMC. Results showed that NR3C1 methylation was significantly higher in ECT non-responders compared to responders at baseline; however, it decreased in both groups after treatment. In contrast, POMC methylation decreased in responders but increased in non-responders by the end of the ECT course. These findings suggest that ECT may induce short-term epigenetic modifications in stress-related genes and that NR3C1 methylation, in particular, could serve as a candidate biomarker for treatment response prediction (39).

Beyond methylation, new studies on small RNA sequencing have provided further insights into the molecular underpinnings of ECT response. In 2023, Kaurani et al. analyzed whole-blood miRNA profiles obtained from 64 patients with treatment-resistant MDD (mean ± SD age: 55.9 ± 16; 59.38% female) at three different times (e.g., before, after the first, and after the last ECT). Clinically, patients were assessed by the MADRS. The main outcome showed that miR-223-3p was down-regulated at baseline among responder subjects (e.g., 62.5% individuals with a reduction of ≥50% in MADRS total score) in comparison to non-responders. A ROC analysis showed that miR-223-3p distinguished these two groups with an area under curve of 0.76 (95% CI = 0.6–0.91; p = 0.0031), supporting its potential as a predictive biomarker. In addition, a negative correlation was observed between miR-223-3p expression and percentage changes in MADRS (r = -0.32; p = 0.04). This finding leads to an understanding that low miR-223-3p expression at baseline correlates with higher severity of depressive symptoms and that its higher post-treatment expression is associated with symptoms improvement. The results of this study also showed higher expressions of proinflammatory markers (NLRP3, IL-6, IL-1B, and TNF-α) at baseline in responders compared to non-responders, again underscoring the potential of ECT for inflammatory modulation (40). Similarly, Galbiati et al. (2025) performed miRNA analyses in 27 TRD individuals (mean ± SD age: 58.1 ± 11.2; 67% female) treated with ECT. Blood samples were obtained at baseline (T0) and 1 month after the last ECT session (T1); depression symptoms were assessed by the MADRS. Their outcomes showed that eight microRNAs were downregulated after ECT. Symptoms improvement among responders (18 out of 27 subjects) directly correlated with changes in miR-30c-5p (r = 0.616; p = 0.006) and miR-324-3p (r = 0.473; p = 0.048) levels between T0 and T1. In the whole sample (e.g., responders and non-responders), only miR-324-3p correlated with depressive symptoms relief (r = 0.460, p = 0.016) (41).

Moreover, mitochondrial function was investigated by Ryan et al. (2023) in a study including 100 individuals with depression (mean ± SD age: 56.36 ± 14.28; 62% female) treated with ECT and 89 healthy controls (mean age 53.42 ± 10.39; 66.29% female). Blood samples were obtained to measure mtDNAcn levels. Assessed outcomes were the depression severity, ECT response, telomere length, and inflammatory markers. The analyses showed significantly elevated mtDNAcn in depressed individuals compared to controls, even after adjustments for potential confounding factors. However, higher baseline mtDNAcn levels were insufficient as a biomarker for ECT outcome prediction, as no significant differences were observed between remitters and non-remitters or responders and non-responders. Alongside this finding, mtDNAcn was not associated with inflammatory markers levels, intensity of depression symptoms, or telomere length (42).

The main characteristics of studies investigating genetic, epigenetic, microRNA, and mitochondrial biomarkers in the context of ECT in mood disorders are shown in **Table 3**.

## Discussion

Our review assessed recent evidence on ECT and mood disorders. The analyzed studies highlight key pathways through which ECT exerts its effects, spanning systemic immune modulation, neuroplasticity, genetic regulation, and mitochondrial function. Despite heterogeneity in studies designs, the collective evidence suggests, in accordance with previous studies of the field, that ECT does not merely act at the neurotransmitter level but induces broader biological adaptations that may underpin its therapeutic efficacy (43). Importantly, these processes are not isolated, as neurotransmitter alterations may act as upstream modulators influencing these multimodal effects.

Regarding systemic inflammation following ECT, some studies reported marked reductions in pro-inflammatory markers such as CRP, IL-6, and TNF-α post-ECT (17, 27, 28), while others revealed a biphasic response, characterized by initial immune activation followed by partial resolution (29, 30). Whether these distinct patterns can reflect consequences of influence by other factors (such as sample profile and assessment methods) or epiphenomenon is still a knowledge gap. While most biomarker studies have emphasized peripheral inflammation, emerging evidence also points to ECT’s capacity to modulate central immune activity. Findings from cerebrospinal fluid suggest that neuroimmune shifts may accompany clinical improvement, supporting a broader view of ECT as influencing both systemic and central pathways (18).

Interestingly, sex-specific immune responses to ECT—such as IL-8 increases associated with symptom improvement only in women—highlight the potential role of sex hormones and sex-linked immune regulation in treatment outcomes (19). Age-related differences have also been noted: adolescents with MDD exhibited a prolonged and sustained reduction in IL-1β and IL-6 post-ECT, alongside an increase in IL-10, an anti-inflammatory cytokine. These findings showed that younger individuals may exhibit a favorable profile in terms of ECT immune-inflammatory modulation, potentially due to greater immune plasticity or a lower baseline inflammatory burden (17). In contrast, among elderly TRD patients, inflammatory markers remained elevated in a subset of individuals, suggesting that, in this population, immune dysregulation may persist despite clinical symptom improvement; it raises concerns about whether residual inflammation contributes to long-term relapse risk (22). The differences between these age groups indicate the potential immune aging (inflammaging) on ECT response (22, 44). Future research should investigate whether baseline inflammatory profiles can predict differential responses to ECT across age groups and explore personalized approaches to optimize treatment efficacy.

Furthermore, specific symptom clusters may also be influenced by immune modulation. Psychomotor symptoms—often disabling and treatment-resistant—have been shown to associate with IL-6 shifts following ECT, suggesting that targeting immune pathways may have functional relevance beyond core mood symptoms (25). In parallel, cognitive vulnerability following ECT may be related to individual inflammatory profiles in patients with MDD. Inflammatory mediators such as NLRP3 inflammasome, IL-18, and NF-κB were significantly elevated after ECT in MDD patients and negatively correlated with memory scores. Similarly, higher pre-treatment TNF-α, IL-10, and CRP predicted poorer cognitive performance during or after ECT in depressed individuals. These findings suggest that pre-ECT immune phenotyping might aid in predicting short-term cognitive risk in this population (23).

Pharmacological strategies targeting neuroinflammation, such as the COX-2 inhibitor celecoxib, have been explored as potential adjuncts in mood disorders treatment (45–48). Indeed, Kargar et al. (2014) demonstrated that celecoxib, compared with placebo, effectively reduced TNF-α levels in patients with BD undergoing ECT. However, no reductions in other inflammatory markers (e.g., IL-6, IL-1, and high-sensitivity CRP) or direct link between TNF-α decrease and greater symptomatic improvement were observed. Specifically on age, a randomized controlled trial including a large sample of cognitively normal individuals with late-life depression showed that either celecoxib or naproxen was insufficient to improve depressive symptoms compared to placebo (47). Additionally, Kargar et al. (2015) explored the effects of celecoxib in manic patients receiving ECT, assessing its potential role in modulating BDNF levels, a critical mediator of neuroplasticity. While there was a trend toward increased BDNF in patients receiving celecoxib, it did not reach statistical significance, and no correlation was observed between BDNF changes and clinical response (48). These findings suggest that while anti-inflammatory strategies may modulate peripheral and central immune-inflammatory responses, their impact on neuroplasticity and symptomatic improvement remains uncertain (45–48).

The neuroplastic effects of ECT have been supported by studies demonstrating increased expression of hippocampal neurogenesis markers (36). These recent findings align with preclinical research showing that electroconvulsive seizures (ECS) in rodents stimulate neural progenitor cell proliferation and dendritic remodeling (49). The absence of significant microglial activation in post-mortem human hippocampal tissue from ECT-treated patients further suggests that ECT may promote structural brain recovery without triggering sustained neuroinflammation (36). However, contrasting results from animal models, such as ECS failing to prevent lipopolysaccharide-induced microglial activation and behavioral effects, reinforce that neuroimmune response to ECT may be context-dependent, varying with baseline inflammatory status and underlying pathology (50). Despite new data, it is still unclear whether neuroplastic changes correlate with long-term functional outcomes. Longitudinal studies assessing whether these biomarkers relate to relapse risk or cognitive effects could refine clinical decision-making; strategies for augmentation of ECT’s neuroplastic effect may further enhance the precision of neuromodulatory interventions. Notably, emerging neuroimaging studies in humans suggest that glial activity may mediate ECT-induced white matter reorganization. Rather than reflecting nonspecific volumetric effects, structural adaptations appear selectively associated with inflammatory shifts in responders, reinforcing the view that glia-mediated immune signaling may support functional plasticity in TRD (35).

Identifying trends in DNA methylation following ECT in the study by Carvalho Silva et al., albeit lacking statistical significance after multiple corrections, suggests possible effects on cellular reprogramming, which may contribute to neuroplasticity and clinical outcomes in MDD (37). This new analysis contributed to pre-existing investigations; however, newer investigations with larger populations are necessary for robust conclusions (7). Beyond global methylation patterns, recent evidence suggests that ECT may epigenetically modulate components of the stress-response system. Changes in the methylation of NR3C1 and POMC—two key HPA-axis genes—were associated with treatment response, indicating that epigenetic regulation of stress-related pathways may represent a mechanistic link between biological vulnerability and ECT efficacy, particularly in stress-linked mood phenotypes (39).

Still within genetics, the valuable findings of two included studies in this review on microRNA among TRD patients treated with ECT (40, 41) fit within the general trajectory of omics research on treatment-resistant depression (51). The findings indicate that biomarker-driven stratification might enable precision-medicine techniques to select appropriate candidates for ECT. In this setting, translational studies showed that antidepressant treatments have been associated with changes in miRNA expression profiles (such as miR-1202) (52), and current research reinforces that interventions targeting miRNAs may regulate immune functions and improve mood disorders treatment outcomes (53–55). However, gaps within the field involving ECT still remain. The stability of DNA methylation and miRNA changes over time, their sensitivity to external factors such as medication use (53, 56), and their specificity to ECT rather than other antidepressant interventions require deep investigation. Longitudinal studies with repeated sampling, larger cohorts, and controlled confounders are essential to determine whether these molecular alterations can be effectively integrated into clinical decision-making.

Mitochondrial function has also emerged as a relevant domain, with one cited study showing elevated mtDNA copy number in MDD patients, though without clear differentiation between ECT responders and non-responders (42). A plausible explanation for the lack of association between mtDNAcn levels and ECT response is that mitochondrial adaptations may occur at a functional rather than a purely quantitative level. While mtDNAcn is an indirect measure of mitochondrial activity, it does not fully capture key parameters such as mitochondrial membrane potential, ATP production, or reactive oxygen species generation (57, 58). The observed variability in mitochondrial adaptations following ECT requires further studies on comprehensive metabolic profiling, particularly in the context of oxidative stress and neuroenergetics (58). Given that mitochondrial dysfunction is implicated in multiple psychiatric disorders, including MDD, BD, and schizophrenia, further exploration of ECT’s role in mitochondrial resilience could refine understanding of its therapeutic potential (59–61). Additionally, interventions that enhance mitochondrial function, such as metabolic modulators or antioxidant therapies, may represent promising adjuncts to ECT, particularly for patients with high oxidative stress burden (58, 60, 61). Despite recent advances, interactions between mitochondrial function, inflammation, and neuroplasticity remain underexplored in mental disorders. Therefore, integrative research assessing these pathways could contribute to new mechanistic concepts.

Beyond ECT, other neuromodulation techniques have been investigated for their effects on immune, inflammatory, and cellular mechanisms in mood disorders. Repetitive transcranial magnetic stimulation (rTMS), transcranial direct current stimulation (tDCS), and vagus nerve stimulation (VNS) have all demonstrated varying degrees of immunomodulatory and neuroplastic effects. A systematic review encompassing both animal and human studies examined the impact of rTMS on inflammatory markers in psychiatric disorders. The review found that while animal models showed positive changes in microglial activity and anti-inflammatory effects associated with behavioral improvement, these findings were not consistently replicated in human studies focusing on TRD. Specifically, several human studies reported rTMS-induced alterations in peripheral inflammatory markers, but only a minority demonstrated an association with clinical treatment response. The review also noted that most studies exhibited poor or moderate quality in bias assessment, indicating a need for more rigorous research in this area (62). A pilot study published in 2024 by Lespérance et al. assessed the immunomodulatory effects of VNS in TRD patients over an extended period of up to four years. Plasma levels of 40 soluble inflammatory molecules, including cytokines, were measured before VNS implantation and throughout treatment. The results showed significant modifications in cytokine levels that were associated with clinical response in depressive symptoms, suggesting a potential relation between inflammatory modulation and response to treatment. Nonetheless, the small sample size and an extended follow-up of this study limits its generalizability (63). A study by Goerigk et al. (2020) examined the role of peripheral biomarkers associated with neuroplasticity and immune-inflammatory processes in patients with BD undergoing tDCS treatment. The findings suggested that tDCS modulates these biomarkers, particularly IL-8, which significantly reduced following active tDCS. Additionally, higher baseline IL-6 plasma levels were associated with symptomatic improvement post-tDCS (64), aligning with ECT and suggesting that pre-existing inflammatory states may influence treatment response (65, 66). Head-to-head comparisons between ECT and other modulatory interventions are needed to delineate their respective neurobiological mechanisms and optimize patient selection among different modalities.

The newer research in this review contributed to the field with noteworthy insights, yet numerous limitations still exist. First, different patients’ profile across samples (e.g., age, sex, clinical history, body mass index, years of education, ethnicity), heterogeneous outcome variables, distinct ECT protocols, serum collections, and time of follow-up may halt the overall interpretation of the included studies. Secondly, the multimodal responses to ECT, even in studies with similar methods, demonstrate the difficulty in identifying standard modulatory mechanisms. Thirdly, while novel results (such as neuroplasticity and genetic/epigenetic findings) TRD mechanistic insights and phenomenological correlations, their clinical translation remains preliminary, necessitating validation in larger cohorts/randomized controlled trials.

In summary, our review support the view that ECT may modulate immune-inflammatory, neuroplastic, and epigenetic mechanisms, reinforcing its function as a systemic neuromodulatory intervention rather than a purely neurotransmitter-based therapy. While these domains have often been studied in isolation, recent findings suggest a complex interplay between immune regulation, synaptic remodeling, and gene expression in underpinning ECT’s therapeutic effects (65). Neuroinflammatory changes may facilitate neuroplasticity by mitigating cytokine-induced synaptic dysfunction and neurodegeneration. This aligns with post-mortem and imaging studies demonstrating hippocampal neurogenesis and structural remodeling in ECT-treated patients (36). Moreover, epigenetic modifications may be molecular switches linking inflammatory modulation to synaptic plasticity (40, 41). Mitochondrial function represents another critical intersection in this neuroimmune interface. Since mitochondria are key oxidative stress and ATP production regulators, their dysfunction could exacerbate neuroinflammation and impair synaptic remodeling. However, current studies suggest that ECT-induced mitochondrial adaptations may occur at a functional rather than a quantitative level, requiring advanced metabolic profiling to elucidate their clinical significance (42, 58).

## Conclusion

Novel research has added important insights into immune-inflammatory, neuroplastic, and genetic mechanisms related to ECT in mood disorders. Despite remaining gaps, these findings pave the way for precision-based therapeutic strategies.

## Glossary

ADARB1: Adenosine Deaminase RNA Specific B1
BD: Bipolar Disorder
BDNF: Brain-Derived Neurotrophic Factor
BPRS: Brief Psychiatric Rating Scale
CAR: Cortisol-to-Albumin Ratio
CGI-S: Clinical Global Impression – Severity Scale
COX-2: Cyclooxygenase-2
CRP: C-Reactive Protein
CRP-albumin ratio: C-Reactive Protein to Albumin Ratio
CYB5B: Cytochrome B5 Type B
DBS: Deep Brain Stimulation
DCX: Doublecortin
DMP: Differentially Methylated Positions
DMR: Differentially Methylated Regions
DNA: Deoxyribonucleic Acid
ECT: Electroconvulsive Therapy
ECS: Electroconvulsive Seizures
EPB41: Erythrocyte Membrane Protein Band 4.1
FAM20C: Family with Sequence Similarity 20 Member C
FDR: False Discovery Rate
GABA: Gamma-Aminobutyric Acid
GFAP: Glial Fibrillary Acidic Protein
HAMD-24: Hamilton Depression Rating Scale 24-item version
HDRS: Hamilton Depression Rating Scale
HMGB1: High Mobility Group Box-1 Protein
Iba1: Ionized Calcium-Binding Adapter Molecule 1
IFN-γ: Interferon Gamma
IL-1β: Interleukin-1 Beta
IL-6: Interleukin-6
IL-8: Interleukin-8
IL-10: Interleukin-10
IL-18: Interleukin-18
IQCE: IQ Motif Containing E
MADRS: Montgomery-Åsberg Depression Rating Scale
MDD: Major Depressive Disorder
MEG: Magnetoencephalography
miRNA: MicroRNA
MLR: Monocyte-to-Lymphocyte Ratio
MMSE: Mini-Mental State Examination
MoCA: Montreal Cognitive Assessment
mtDNAcn: Mitochondrial DNA Copy Number
NF-κB: Nuclear Factor Kappa B
NLR: Neutrophil-to-Lymphocyte Ratio
NLRP3: NLR Family Pyrin Domain Containing 3 Inflammasome
NR3C1: Nuclear receptor subfamily 3 group C member 1 (glucocorticoid receptor)
OTUB1: OTU Deubiquitinase, Ubiquitin Aldehyde Binding 1
PDGF-BB: Platelet-Derived Growth Factor Subunit B
PDW: Platelet Distribution Width
PLR: Platelet-to-Lymphocyte Ratio
POMC: Proopiomelanocortin
PVRL4: Poliovirus Receptor-Related 4
QoL: Quality of Life
RDW: Red Cell Distribution Width
rTMS: Repetitive Transcranial Magnetic Stimulation
sRAGE: Soluble Receptor for Advanced Glycation End Products
SD: Standard Deviation
SII: Systemic Immune-Inflammation Index
STMN1: Stathmin 1
tDCS: Transcranial Direct Current Stimulation
TNF-α: Tumor Necrosis Factor Alpha
TRD: Treatment-Resistant Depression
TR-MDD: Treatment-Resistant Major Depressive Disorder
VNS: Vagus Nerve Stimulation
WMS-R: Wechsler Memory Scale–Revised
WHOQOL-BREF: World Health Organization Quality of Life – Brief Version
